# Editorial: Antipsychotics of New Generation: Where Are We now?

**DOI:** 10.3389/fphar.2021.646286

**Published:** 2021-02-22

**Authors:** Tatiana V. Lipina, Colm O'Tuathaigh, Shupeng Li

**Affiliations:** ^1^ Dementia Research Center, University College London, London, United Kingdom; ^2^ University College Cork, Cork, Ireland; ^3^ Peking University, Beijing, China

**Keywords:** schizophrenia, genetics, hippocampal neural circuits, neurodevelopment, pharmacology, animal models

**FIGURE 1:**
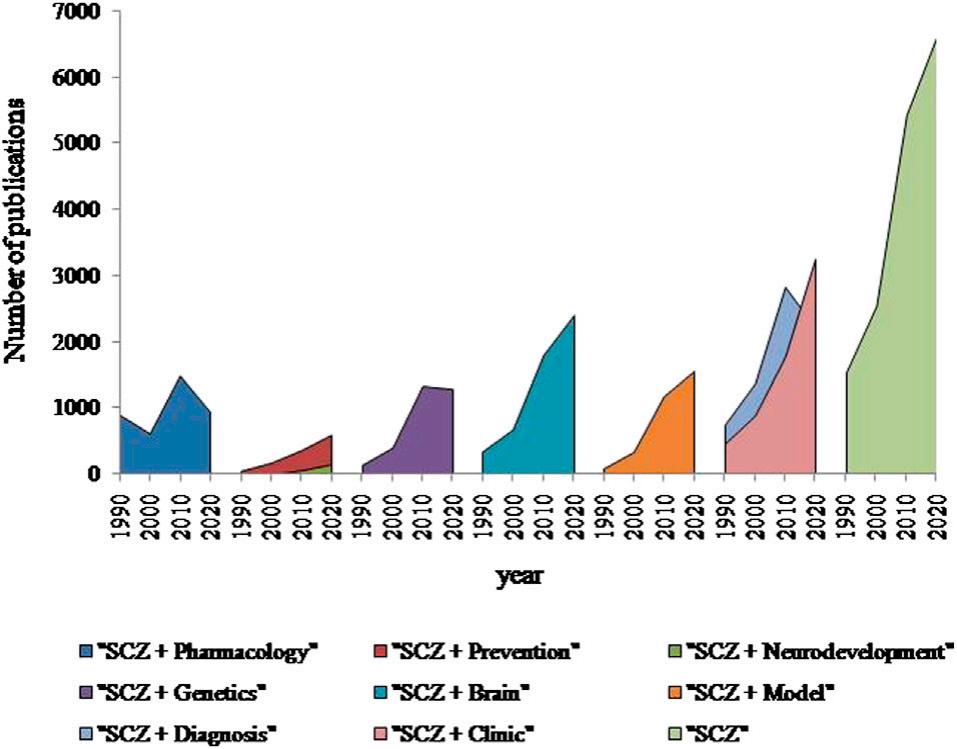
Number of publications found in PubMed for every 10th year since 1990 using keywords indicated in the legend (Schizophrenia (SCZ)). Although the amount of studies related to schizophrenia was increased in 4.3 times since 1990, there is a clear reduction of the pharmacological studies related to schizophrenia in the last decade. However, schizophrenia research dedicated to the brain, diagnosis, clinical studies or animal models has a constant growth during last 4 decades. Notable, that research on neurodevelopment and preventive therapy appears as an emerging direction in the field.

Schizophrenia is a complex mental disorder affecting 0.3–0.7% of people (Javitt, 2014), causing severe psychotic episodes which lead to patient disability. The progress of modern tools in the field of neuroscience has been applied to schizophrenia research and significantly increased number of published papers during the last 40 years (Figure 1). Indeed, schizophrenia research dedicated to neuroimaging of brain, molecular-genetics clinical studies or development of animal models shows a stable increase during the last 4 decades. Moreover, research on neurodevelopment and preventive therapy appears as an emerging and promising direction in the field. In contrast, pharmacological studies show modest reduction during the last 10 years, reflecting a high percentage of failures in drug discovery for several reasons as discussed (e.g., Griebel, 2017). Even schizophrenia research is providing an increasing number of studies and important insights about aetiology of this mental disorder based on genetics, neuroimaging or preclinical studies, the effective strategies to incorporate advanced findings into clinics like RDoC framework (Sanislow et al., 2019) is still on their way. The inevitable challenge in the clinic of schizophrenia will be an elaboration of comprehensive diagnostic tools to facilitate the advances in drug discovery of antipsychotics of a new generation.

Ten years ago Thomas Insel has asked: “How we will view schizophrenia in 2030?” (Insel, 2010) where presented and discussed schizophrenia as a neurodevelopmental disorder with a big hope for its prevention and treatment in near future. Indeed, some progress was achieved to deeper understand molecular-cellular, biochemical and neurobiological mechanisms of schizophrenia during its neurodevelopment. For instance, the DISC1 (Disrupted-in-Schizophrenia-1) studies have convincingly made great progress in this directing, discovering the role of DISC1 and its interactome in neuronal proliferation, migration, integration and maturation, interaction with pathogenic environmental factors, triggering schizophrenia (Lipina and Roder, 2014). Waddington with co-authors reviewed in details the current progress on schizophrenia and neurodevelopment (Waddington et al.). Dysbindin-1 promotes cellular and intracellular processes related to cellular growth and proliferation via Akt. Dysbindin forms a molecular complex with BLOC-1, pallidin, MUTED and snapin, where Dysbindin x MUTED increased the cell surface of D2 receptors, whereas BLOC-1 interacts with DISC1 and SNARE, affecting dopaminergic and neurodevelopmental functions. Several potential drug targets for antipsychotics have emerged based on dysbindin1-related clinical and animal studies, including D3R; NKCC1 and its antagonist, bumetanide reduced hallucinations in humans; sphingosine1-phosphate receptor and its modulator increased BDNF expression and improved recognition memory and socializing, whereas mGluR5 and its positive modulator, CDPPB corrected cognitive phenotypes in sdy/B6 mice.

Fast development and integration of such new methods in neuroscience as e.g. optogenetics with spanning electrophysiology or Ca^2+^-imaging allow accurately detect neural network on cellular, circuit-level or brain-wide scales. The hyperactivity of the hippocampus was detected as a robust correlate of schizophrenia at the early-life stage by imaging clinical studies. Kätzel with co-authors hypothesized the hippocampal hyperactivity as a druggable circuit-level target to regulate aberrant salience as an important endophenotype of schizophrenia (Kätzel et al.). Hallucinations and delusions are the main positive symptoms of schizophrenia. The term “aberrant silence” suggests that delusions are the result of the brain’s attempt to make sense of a neural representation of a world where erroneously high significance is assigned to certain items. Schizophrenics showed higher aberrant salience, which is associated with the severity of positive symptoms. Prodromal patients at ultra-high risk of developing schizophrenia showed increased aberrant silence. Kätzel with co-authors comprehensively reviewed the accumulated fMRI clinical findings coupled with psychological assessments to outline the implication of dopaminergic midbrain regions (VTA, SNc), the amygdala, anterior cingulated cortex, parahippocampal gyrus, striatum and cerebellum into the regulation of the aberrant silence. Authors emphasized that early interventions that modulate neural circuits controlling the dopaminergic system are a promising direction that could lead to the development of antipsychotic of a new generation. Hence, the upstream circuits regulating dopaminergic functions of the hippocampus were further reviewed. Independent evidence indicates that the hippocampus contributes to the computation of the saliency of specific stimuli based on their novelty/familiarity in reward-/ and sensory experience-dependent manners. The groundbreaking hypothesis provided several molecular targets that can be pharmacologically treated to reduce the hippocampal hyperactivity, correct the increased aberrant silence. Overall, Kätzel suggested the promising strategy for the drug discovery in schizophrenia “from brain area to molecular target via specific cellular sub-population”.

In principle, the same strategy can be applied to other schizophrenia phenotypes. Alexandrov with colleagues focused in their pre-clinical experimental study on effects of TAAR1 agonist on the mismatch negativity (MMN) as a promising and sensitive tool in pre-clinical psychiatry phenotype of schizophrenia (Alexandrov et al.) and awaits the detection of new molecular-cellular targets underlying MNN.

Given that translation of pre-clinical knowledge requires significant efforts to incorporate it in clinics, it is a high priority to optimize the application of currently available pharmacological drugs. The clinical study led by Zai probed association between the copy numbers of the complement component 4 (C4)-A and B-gene with tardive dyskinesia induced by typical antipsychotics (Zai et al.). Although the study had several limitations, authors detected a significant link between the copy number of structural element C4BL and severity of tardive dyskinesia, implicating to the development of personalized medication of schizophrenia (Foley et al., 2017). Another clinical study designed by Baltzersen with colleagues aimed to identify specific “serotonergic subtype” of schizophrenics by utilizing efficacy of Pimavanserin (5-HT2AR antagonist) on schizophrenia assessed by the Cambridge Neuropsychological Test Automated Battery and neuroimaging techniques, which will contribute to the personalized medicine in psychiatry (Baltzersen et al.).

Taken together, growing studies dedicated to the neurodevelopment, neural circuitries, or genetics will provide a solid fundament to facilitate diagnostics, prevention and treatment of schizophrenia. Nevertheless, pharmacology of schizophrenia is facing the imminent challenges at the end of 2020 with an urgent need to effectively integrate translatable knowledge from the neuroscience and comprehensive clinical studies to create antipsychotics of a new generation.
